# Multiomics insights into quinolizidine alkaloid biosynthetic architecture in narrow-leafed lupin genotypes with contrasting alkaloid regulation

**DOI:** 10.1093/hr/uhag232

**Published:** 2026-06-08

**Authors:** Katarzyna Czepiel, Aleksandra Burdzińska, Karolina Susek, Agnieszka Kiełbowicz- Matuk, Grzegorz Koczyk, Magdalena Kroc

**Affiliations:** Legume Genomics Team, Institute of Plant Genetics, Polish Academy of Sciences, Poznań 60-479, Poland; Multiomics Laboratory, Institute of Plant Genetics, Polish Academy of Sciences, Poznań 60-479, Poland; Legume Genomics Team, Institute of Plant Genetics, Polish Academy of Sciences, Poznań 60-479, Poland; Regulation of Gene Expression Team, Institute of Plant Genetics, Polish Academy of Sciences, Poznań 60-479, Poland; Biometry and Bioinformatics Team, Institute of Plant Genetics, Polish Academy of Sciences, Poznań 60-479, Poland; Legume Genomics Team, Institute of Plant Genetics, Polish Academy of Sciences, Poznań 60-479, Poland

## Abstract

Quinolizidine alkaloids restrict the use of lupin seeds as food and feed. In narrow-leafed lupin, low-alkaloid content in most cultivars has been traced to the recessive *iucundus* locus, with the RAP2-7 transcription factor as a candidate regulator. The molecular basis of alternative low-alkaloid sources remains unclear. The aim of this study was to elucidate the molecular determinants underlying low-alkaloid content through a comparative analysis of the classical *iucundus* and alternative Bryansk backgrounds, with particular emphasis on the molecular characterization of *RAP2-7*. This multiomic framework integrates alkaloid profiling and transcriptomics alongside RAP2-7 DNA binding characterization, and sequence-level motif and variant analyses. Alkaloid profiles revealed genotype-specific differences, with Bryansk lines showing a distinct, sparteine-enriched and lupanine-depleted chemotype relative to *iucundus* and *Iucundus*. Using *Iucundus* line as a reference, transcriptome analyses highlighted candidate genes associated with low-alkaloid *iucundus* and Bryansk lines, spanning enzymes, transporters and putative regulators. Consistent with a key role of RAP2-7, DAP-seq summits in *Iucundus* and Bryansk contained a clear AP2-like motif, whereas the *iucundus* background showed both strong depletion of high-confidence peaks and no defined motif. *In silico* modelling of RAP2-7 bound to its DNA motif, combined with DAP-seq and expression data, supported reduced binding of the *iucundus* variant relative to *Iucundus,* as well as a possible role for a mutation within the promoter of key acyltransferase *LaAT*. Collectively, these data extend lupin transcriptomic resources and refine models of alkaloid biosynthesis beyond the classical *iucundus* background, while also providing the first comprehensive molecular characterization of Bryansk low-alkaloid lines.

## Introduction

Plants synthesize a diverse spectrum of specialized metabolites that often contribute to their ecological fitness and adaptive potential. In lupins, quinolizidine alkaloids (QAs) form a major class of such compounds, underpinning plant defense and shaping end-use quality of seeds [1]. Given their toxicity, feed safety requires QA content below ~0.02% of seed dry weight. Corresponding genotypes with reduced QA accumulation are classified as low-alkaloid or sweet genotypes, in contrast to high-alkaloid, or bitter lines [2].

A comprehensive understanding of a crop trait requires elucidation of its molecular basis, including the underlying pathway, regulation and transport, together with characterization of genetic resources to capture allelic diversity and relevant breeding sources. In lupins, studies of QA biosynthesis have mapped parts of the pathway, including a few enzymes and their organ specific expression patterns, yet the pathway as a whole remains incompletely understood, with recent mechanistic considerations suggesting several steps lacking identified catalytic enzymes [3–15]. Moreover, seed QA profiles in lupin germplasm have been partly characterized, revealing variation relevant to breeding [16–20]. Here, we focus on narrow-leafed lupin (*Lupinus angustifolius* L., NLL), where low seed QA may derive from several genetic factors [16]. Among these, the recessive *iucundus* locus is a naturally occurring mutation first described in the early 20th century [21], that later became a widely used source of low QA in NLL breeding programs [22], although the gene underlying this locus remained unknown for decades. In our previous work [11, 12], *RAP2-7* was identified as a candidate gene co-segregating with *iucundus* and was proposed as a key regulatory factor in QA biosynthesis. As a member of the AP2/ERF transcription factor (TF) family, previous phylogenetic analyses indicated its relationship to the Arabidopsis flowering repressors TOE2, SMZ and SNZ [12]. Additionally, in the NLL collection, the R196S substitution in exon 4 of *RAP2-7* was strongly associated with reduced seed QA content, suggesting a partial reduction in RAP2-7 activity tied to *iucundus* allele [11]. Interestingly, in the same study, we identified three low-QA lines carrying the *RAP2-7* allele associated with the high-alkaloid phenotype and exhibiting high expression of known QA-related genes. These observations indicated that the lines, originating from Bryansk and hereafter referred to as the “Bryansk lines,” likely harbor an alternative locus controlling low QA content in seeds, distinct from *iucundus* [11, 12]. Despite these advances, the molecular basis of RAP2-7 regulatory role in QA biosynthesis still requires clarification. Likewise, the molecular determinants of the low-QA phenotype in the Bryansk lines have yet to be elucidated.

Plant alkaloid research increasingly uses complementary datasets that combine organ resolved alkaloid and expression profiles with genome assemblies, single cell datasets, and long-read resources that refine gene model. Such integrative strategies have supported pathway reconstruction, enzyme and transport assignment, gene cluster detection, and candidate biosynthetic genes selection in several systems, i.e. for monoterpenoid indole alkaloids (MIA) in *Catharanthus roseus* L. [23–27] and benzylisoquinoline alkaloids (BIA) in opium poppy [28–30]. To fully interpret plant alkaloid pathways, alongside biosynthetic reconstruction, the regulatory layers need to be resolved. DNA affinity purification sequencing (DAP-seq) is a promising approach that enables genome-wide *in vitro* mapping of transcription factor binding sites (TFBS) and serves as an antibody-free alternative to ChIP-seq [31, 32]. This approach, novel in lupin, facilitates investigation of TF binding sites underlying regulatory networks for key agronomic traits, including alkaloids.

In this work, we investigated two genetic backgrounds underlying QA regulation in NLL: the *iucundus* locus contrast, represented by 83A:476 (*iuc*, low-alkaloid) and P27255 (*Iuc*, high-alkaloid), and an alternative low-alkaloid regulatory type represented by the Bryansk lines (95826, 95927, and 95928) (see Materials and Methods for details). Using an integrated omics framework, we addressed two specific questions: how these two genetic architectures differ in the regulation of QA biosynthesis, and how the genome-wide binding profile of RAP2-7 ties into its role in transcriptional regulation in NLL. To characterize these contrasts, we profiled QA content, composition, and the expression of pathway genes across multiple organs to define their genetic and regulatory features. Complementary long-read and short-read RNA sequencing of leaves and stems refined gene annotations and revealed genotype-specific expression differences. Finally, allele specific RAP2-7 binding patterns across the NLL genome in both regulatory contrasts were characterized using DAP-seq. Notably, this work provides the first integrated molecular characterization of the Bryansk regulatory type. Together, these datasets uncover the multilayered and genotype-specific nature of QA biosynthesis in lupin, provide an integrated framework to compare established and alternative mechanisms leading to low seed QA, refine the working model of RAP2-7 function, and prioritize candidates for functional validation.

## Results

### Alkaloid quantification in lupin organs

#### Total alkaloid content

Total alkaloid content (TAC) varied strongly between organs and genotypes (Table 1). Bryansk lines exhibited markedly lower TAC than P27255, but higher than 83A:476. Differences were greatest in leaves, with the Bryansk lines containing 10–25× more alkaloids than 83A:476, while remaining well below P27255 (Table 1). A similar pattern was observed in stems, flowers and pods, with TAC above 1% in *Iucundus* and below 0.1% in the Bryansk lines. TAC increased from vegetative to reproductive organs (except pods), reaching the highest values in seeds. Notably, the Bryansk lines exceeded 83A:476 by three to five-fold in green seeds, while in dry seeds both were comparable and well below P27255.

**Table 1:** Total alkaloid content (TAC, % dry weight) across organs of NLL in *iucundus* (83A:476), *Iucundus* (P27255), and Bryansk lines (95826, 95927, 95928).

Organ	TAC (% of dry weight)				
	83A:476	P27255	95826	95927	95928
Leaves	0.0017	0.7790	0.0448	0.0178	0.0198
Stems	0.0298	1.1219	0.0582	0.0452	0.0391
Flowers	0.0176	3.7059	0.0884	0.0946	0.0443
Green pods	0.0112	1.0373	0.0297	0.0230	0.0224
Green seeds	0.0333	2.9495	0.1585	0.1841	0.1148
Dry seeds	0.0648	2.3891	0.1354	0.0905	0.0746

Organs as defined in section Materials and Methods (Table 4).

#### Distribution of individual alkaloids across plant organs

Qualitative QA composition varied by genotype and organ, with the Bryansk lines showing broadly similar profiles (Fig. 1; Table S1). Lupanine dominated in P27255 (~80%), and was moderate in 83A:476 and the Bryansk lines (~30%–40%). 13-Hydroxylupanine was slightly higher in the Bryansk lines and 83A:476 (~25%–30%) than in P27255 (~15%) and, in the Bryansk lines, showed an accumulation trend opposite to the other genotypes, increasing from leaves to seeds. Isolupanine was most abundant in the Bryansk lines (16%–19%) and lowest in P27255 (3%), typically decreasing toward seeds. Angustifoline was inconsistently detected, and most abundant in dry seeds of the Bryansk lines and 83A:476. Notably, the Bryansk lines accumulated much more sparteine (~20%) than P27255 and 83A:476 (~0.5%), except in 83A:476 pods. Other alkaloids were low and infrequent, with P27255 displaying the broadest spectrum consistent with its high TAC, whereas 83A:476 was distinguished by substantial multiflorine accumulation in stems and seeds.

**Figure 1:**
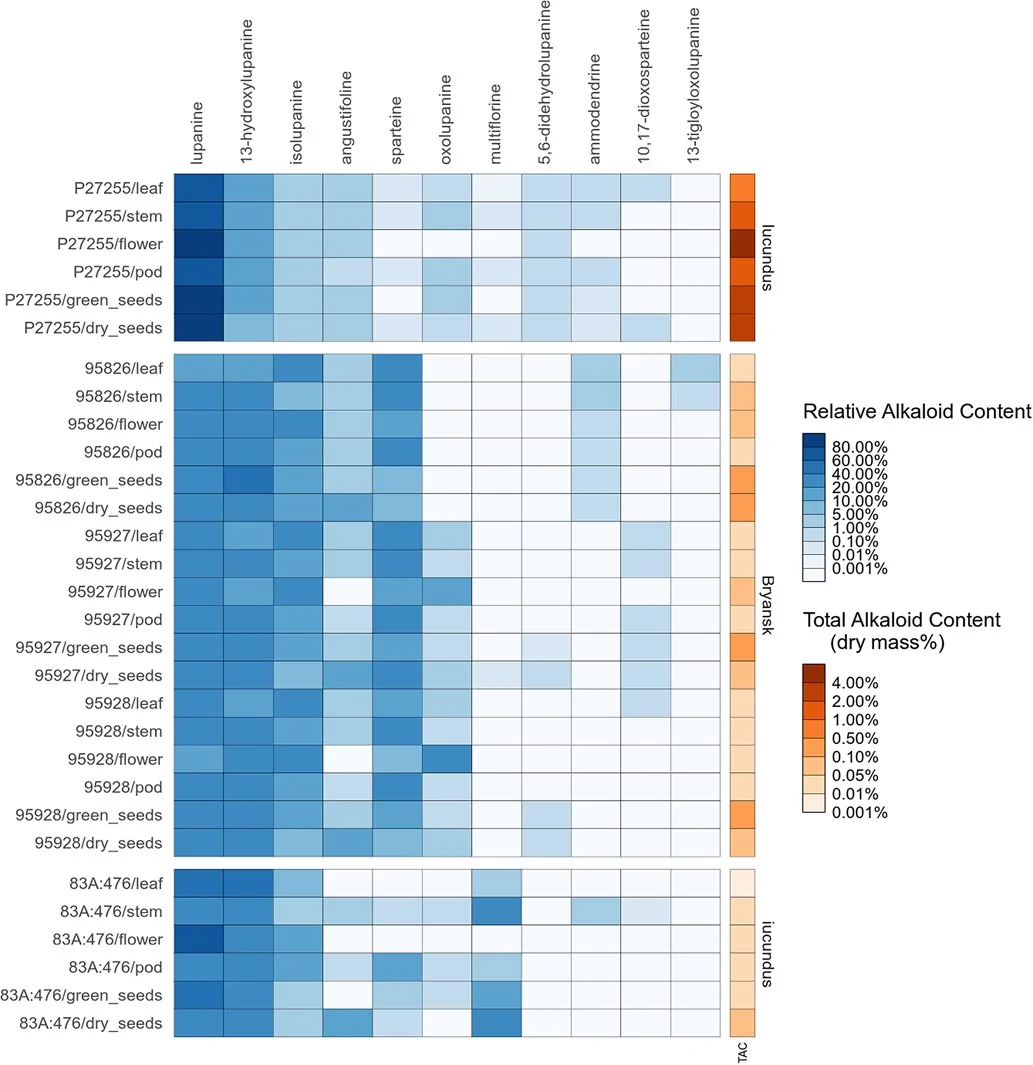
Heatmap of organ resolved QA profiles in NLL assessed by gas chromatography. Each cell shows the percent contribution of a compound to total alkaloids in a sample. The adjacent bar indicates total alkaloid content (percent dry mass). Values are means of three biological replicates.

### Organ-specific expression of QA-related genes across regulatory types

Across all genes examined, expression differences between P27255 and 83A:476 references were most evident in leaves and pods (Fig. 2). Stems and flowers did not show clear or consistent separation between the two genotypes, while expression in seeds was low or variable. In the Bryansk lines expression of the assayed genes was comparable to that observed in P27255. Overall, in P27255 and the Bryansk lines, expression of the assayed genes was most pronounced in green aerial lupin organs (leaves, stem, and pods), with *LDC* showing the highest and *RAP2-7* generally the lowest relative expression among the analyzed genes.

**Figure 2:**
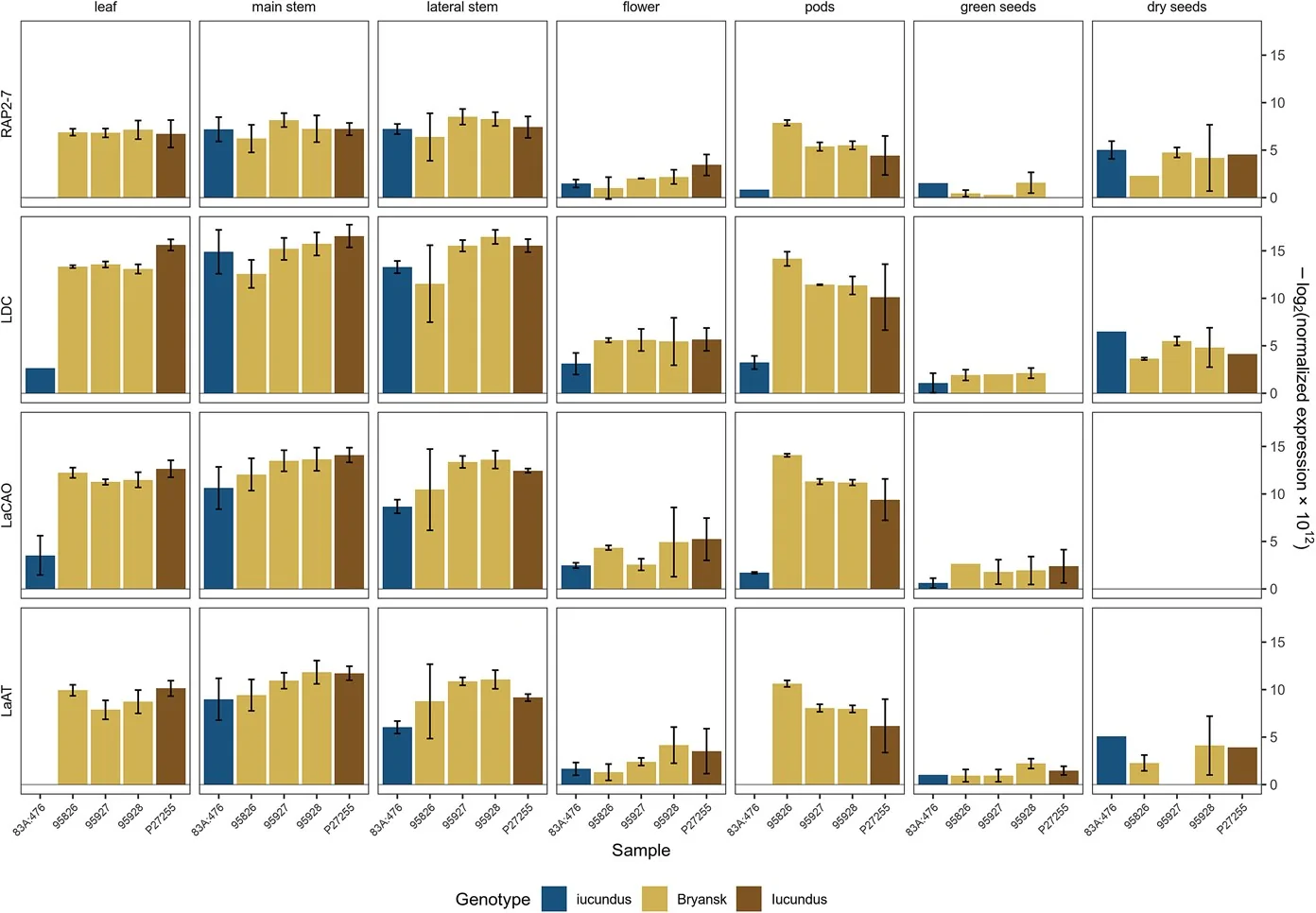
Organ-specific expression of genes involved in QA pathway in five NLL genotypes, assessed by qPCR. Bar plots show mean normalized expression with standard error. Bar colors indicate genotypes grouped as *iucundus*, Bryansk lines and *Iucundus* as shown in the legend.

### NLL transcriptome analysis using PacBio Iso-Seq and RNA-Seq

Comprehensive NLL transcriptome characterization was achieved by integrating PacBio Iso-Seq and Illumina RNA-seq for leaves and stems of analyzed genotypes (Tables S2–S4). PacBio libraries (one per genotype and organ) produced 14.2–22.3 M subreads for leaves (33.1–57.0 Gb) and 14.6–77.4 M (33.7–160.8 Gb) for stems. After SQANTI3 filtering, the clustered Iso-Seq transcriptome comprised 19 071 genes and 100 203 transcripts (N50 2839 bp), increasing to 38 236 genes and 119 368 transcripts after adding gene models lifted from the NLL references [33, 34] to recover loci not captured by the long-read data. The refined locus catalogue is summarized in Tables S5–S7, which describe its composition after integrating PacBio Iso-Seq (PB) with gene models lifted from v1 [34] and v2 [33], quantify the overlap between PB loci and each annotation release, and provide mapping across all three identifier categories. As shown in the cross-reference table (Table S6), 95.7% of the PB loci can be linked to identifiers in both annotation releases, indicating that PacBio Iso-Seq provided independent, full-length transcript evidence that refined transcript structures for previously annotated loci, thereby improving the Iso-Seq transcriptome resource. Illumina libraries from leaves and stems yielded 82.0–102.9 million reads per genotype library (12.3–15.4 Gb per sample), with more than 97% of bases above Q20 (Table S2). Relative to this, a merged transcriptome based on the previously published genome annotations v1 [34] and v2 [33] comprises 39 130 genes/transcripts (N50 1701 bp) (Table S4). Functional annotation assigned at least one GO term to 30 089 of the 38 236 genes (78.7%; Table S8).

### Characterization of differentially expressed genes and candidate genes selection

Across all analyses, 18 851 genes met the significance threshold (Table S9). Separate DEG sets were generated for leaves and stems in *iuc* vs *Iuc* and Bryansk vs *Iuc* contrasts (Tables S10–S13). The high-confidence consensus differentially expressed gene (DEG) set comprised genes identified by all three methods (see Materials and Methods) and was used for subsequent analyses. In both contrasts leaves contributed more consensus DEGs than stems, comprising 663 DEGs in leaves (Table S12) and 315 in stems (Table S13) in *iuc* vs *Iuc*, and 622 in leaves (Table S10) and 352 in stems (Table S11) in the Bryansk vs *Iuc*.

Gene Ontology enrichment of the consensus DEGs showed that induced or suppressed biological processes differed, depending on both contrast and organ (Fig. 3; Table S14). Induced processes were mostly shared in leaves and stems of *iucundus* and the Bryansk lines compared with *Iucundus*, whereas the repressed processes generally differed between both contrasts. In leaves, *iucundus* line showed reduced defense and hormone responses, as well as decreased transmembrane transport and catabolic processes. Bryansk leaves instead were depleted in responses to other organisms and toxic substances as well as in xenobiotic and amino acid transport, as well as hormone related processes. In stems, down-regulation in *iucundus* mainly affected flavonoid and anthocyanin biosynthesis and auxin and purine transport, while in Bryansk lines it primarily involved late endosome to vacuole transport. Shared induced categories in both organs of *iucundus* and the Bryansk lines included hydrogen peroxide catabolism, response to oxidative stress, responses to host organisms and defense mechanisms of other organisms, cellular development, antibiotic metabolism, lysine biosynthesis via diaminopimelate, cytokinin responses and Golgi vesicle budding. Additional enriched categories were also noted in particular organs or genotypes as detailed in Table S14.

**Figure 3:**
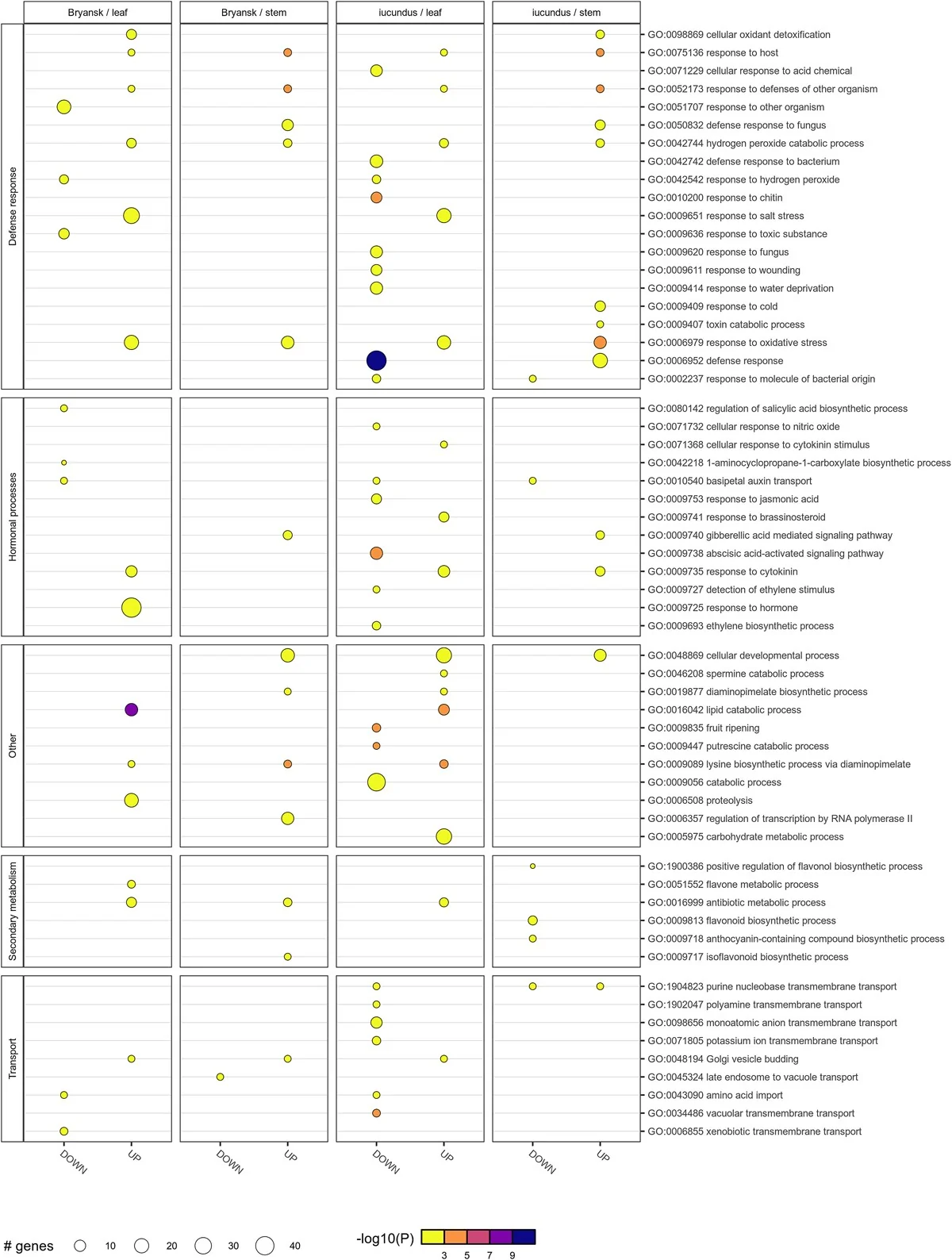
Gene ontology biological process enrichment for DEGs in leaves and stems of Bryansk lines and *iucundus* relative to *Iucundus*. Bubble plot showing enriched Gene Ontology biological process terms. Columns subdivide data by contrast/organ combinations with upregulated and down-regulated genes shown separately. Bubble size reflects the number of significant DEGs associated with each term and color indicates enrichment significance expressed on the minus log10 P-value scale.

To examine QA-related processes, further analyses focused on the down-regulated gene sets from both contrasts, comprising 259 and 96 genes in leaves and stems for the *iuc* vs *Iuc*, and 168 and 80 for the Bryansk vs *Iuc*. In total, 53 candidate genes were selected, including 17 from the *iuc* vs *Iuc* contrast, 29 from the Bryansk vs *Iuc* contrast, and seven known QA-related genes serving as a reference set (Table 2). For chromosomal context, candidate genes from the *iuc* vs *Iuc* contrast, together with the known QA-related genes were mapped onto the *iucundus* reference genome [33] (Fig. 4). These genes were widely distributed across the genome, spanning 13 of the 20 NLL chromosomes, with the highest number on NLL-15, and a notable local cluster of three MLP423 genes on chromosome NLL-06.

**Table 2:** Candidate genes involved in QA pathway with functional annotation and differential expression metrics in *iucundus* vs *Iucundus* and Bryansk vs *Iucundus* contrasts.

Candidate gene set	Functional category	Gene ID (current)	Gene symbol	Gene IDs in previous annotations (v1, v2)	Gene annotation (NCBI RefSeq, *Lupinus angustifolius*)	Linkage group (NLL)	DEG log2FC (E-value, method)			
							***iucundus* vs *Iucundus*, leaves**	***iucundus* vs *Iucundus*, stems**	**Bryansk vs *Iucundus*, leaves**	**Bryansk vs *Iucundus*, stems**
*iucundus* vs *Iucundus* contrast	Secondary metabolism and biosynthesis	PB.16323	***ANR***	TanjilG_14005, Lupan_020294	Anthocyanidin reductase ((2S)-flavan-3-ol-forming)-like	NLL-15	−12.64 (3.99e-19; **S**)	0	0	0
		Lupan_007209	***CES1L***	TanjilG_32669, Lupan_007209	Carboxylesterase 1-like	NLL-08	−5.52 (1.10e-13; **S**)	0	0	0
		PB.14959	***LDOX***	TanjilG_00883, Lupan_021727	Leucoanthocyanidin dioxygenase-like isoform X2	NLL-18	−23.03 (8.75e-09; **S**)	−34.56 (2.06e-20; **S**)	0	0
		PB.2970	***MLP31_1***	TanjilG_01236, Lupan_002572	MLP-like protein 31	NLL-06	−15.38 (7.42e-32; **S**)	0	0	0
		PB.20574	***MLP31_2***	TanjilG_26043, Lupan_028507	MLP-like protein 31	NLL-10	−5.62 (1.45e-09; **R**)	0	0	0
		Lupan_028512	***MLP31_3***	TanjilG_19334, Lupan_028512	MLP-like protein 31	NLL-10	−13.91 (2.28e-28; **S**)	0	0	0
		PB.2036	***MLP423_1***	TanjilG_15923, Lupan_000661	MLP-like protein 423 isoform X1	NLL-06	−14.86 (3.11e-29; **S**)	0	0	0
		PB.2037	***MLP423_2***	TanjilG_15922, Lupan_000662	MLP-like protein 423 isoform X2	NLL-06	−13.40 (8.40e-20; **S**)	0	0	0
		PB.2039	***MLP423_3***	TanjilG_15921, Lupan_000663	MLP-like protein 423	NLL-06	−8.03 (1.88e-14; **R**)	0	0	0
		Lupan_011464	***N8DT-2***	TanjilG_21382, Lupan_011464	naringenin 8-dimethylallyltransferase 2, chloroplastic-like isoform X4	NLL-04	−6.94 (3.35e-11; **R**)	0	−4.82 (9.50e-09; **R**)	0
		PB.19347	***VR1***	TanjilG_05322, Lupan_030549	Vestitone reductase-like	NLL-14	−7.26 (2.00e-12; **S**)	0	0	0
		PB.19348	***VR2***	TanjilG_05321, Lupan_030550	Vestitone reductase-like	NLL-14	−6.05 (3.10e-10; **S**)	0	0	3.08 (2.15e-03; **k**)
	Transcription and gene expression regulation	PB.16358	***MYB34***	TanjilG_10046, Lupan_036882	MYB34 TF	NLL-15	−8.76 (1.12e-05; **S**)	0	0	0
		PB.20273	***RAP2–7A***	TanjilG_14185, Lupan_023724	Ethylene-responsive TF RAP2–7-like	NLL-13	−10.02 (2.42e-13; **S**)	0	0	0
	Metabolite transport	PB.16702	***PUP1***	TanjilG_28431, Lupan_020878	Purine permease 1-like	NLL-15	−12.05 (1.54e-23; **S**)	−3.27 (4.02e-04; **k**)	0	0
		PB.17620	***SLC35F2***	TanjilG_27659, Lupan_028023	Solute carrier family 35 member F2-like	NLL-19	−23.40 (3.23e-15; **R**)	0	0	0
	Other	Lupan_007816	**Lupan_007816**	TanjilG_08196, Lupan_007816	Uncharacterized protein LOC109342541 isoform X1	NLL-03	−9.12 (2.83e-08; **S**)	0	0	0
*Bryansk vs Iucundus* contrast	Protein sorting and organelle trafficking	PB.3562	***BLOS1***	TanjilG_24054, Lupan_032439	Biogenesis of lysosome-related organelles complex 1 subunit 1-like isoform X1	NLL-11	0	0	−4.05 (1.68e-65; **k**)	−5.17 (1.29e-122; **k**)
		Lupan_002219	***CHMP1B***	TanjilG_05990, Lupan_002219	ESCRT-related protein CHMP1B-like	NLL-06	0	0	−12.24 (2.37e-88; **S**)	−11.90 (7.20e-83; **S**)
		Lupan_032803	***IST1***	TanjilG_15149, Lupan_032803	IST1 homolog [*Arachis ipaensis*]*	NLL-11	−2.89 (1.98e-03; **k**)	0	−3.20 (3.12e-07; **k**)	−3.44 (2.52e-04; **R**)
	Transport and ion homeostasis	PB.9831	***CAT***	TanjilG_15405, Lupan_009525	Cationic amino acid transporter 7, chloroplastic-like	NLL-04	−3.17 (4.42e-03; **k**)	−3.82 (5.95e-05; **k**)	−3.44 (9.91e-06; **k**)	−2.90 (2.04e-03; **k**)
		PB.8060	***CAX3***	TanjilG_24316, Lupan_013395	Vacuolar cation/proton exchanger 3-like	NLL-07	−6.34 (8.33e-08; **S**)	0	−5.54 (1.67e-08; **S**)	0
		Lupan_015411	***CNGC1***	TanjilG_02509, Lupan_015411	Cyclic nucleotide-gated ion channel 1-like isoform X2	NLL-02	−2.90 (1.93e-03; **k**)	0	−2.47 (3.99e-03; **k**)	0
		Lupan_018450	***DTX16***	TanjilG_02143, Lupan_018450	Protein DETOXIFICATION 16-like isoform X2	NLL-05	0	−2.19 (5.04e-04; **k**)	−2.13 (8.26e-06; **k**)	−2.69 (8.57e-13; **k**)
		Lupan_008507	***DTX21***	TanjilG_00065, Lupan_008507	Protein DETOXIFICATION 21-like	NLL-03	−5.06 (1.32e-07; **k**)	0	−5.17 (2.01e-12; **k**)	0
		PB.21510	***KEA18***	TanjilG_25011, Lupan_024380	Cation/H(+) antiporter 18-like	NLL-17	−4.32 (1.98e-07; **R**)	−4.13 (1.21e-06; **R**)	−4.15 (2.08e-10; **R**)	−3.41 (3.95e-06; **k**)
		PB.2596	***NPF6.3***	TanjilG_32231, Lupan_001540	protein NRT1/PTR FAMILY 6.3-like	NLL-06	−3.44 (1.14e-04; **S**)	0	−2.57 (8.99e-04; **R**)	0
		PB.19119	***WAT1***	TanjilG_12690, Lupan_030170	WAT1-related protein At4g08300-like	NLL-14	−4.29 (1.47e-08; **R**)	0	−3.04 (2.44e-05; **R**)	0
		Lupan_007045	***ZIFL1***	TanjilG_22335, Lupan_007045	Protein ZINC INDUCED FACILITATOR-LIKE 1-like isoform X5	NLL-08	−3.95 (1.02e-72; **S**)	0	−3.26 (3.22e-81; **k**)	0
	Ethylene biosynthesis	PB.12627	***ACO4***	TanjilG_24114, Lupan_026415	1-aminocyclopropane-1-carboxylate oxidase homolog 4-like	NLL-09	−3.81 (3.56e-10; **k**)	0	−2.98 (1.23e-07; **k**)	0
		PB.14361	***ACS***	TanjilG_04889, Lupan_022502	1-aminocyclopropane-1-carboxylate synthase-like	NLL-18	0	0	−4.77 (1.47e-08; **R**)	0
	Transcription and gene expression regulation	PB.6812	***AP2_41710***	TanjilG_26837, Lupan_019639	AP2-like ethylene-responsive-responsive TF At2g41710	NLL-05	0	0	−1.96 (1.28e-03; **R**)	0
		PB.16404	***bHLH137***	TanjilG_25129, Lupan_020419	TF bHLH137-like isoform X1	NLL-15	0	0	−2.55 (7.45e-04; **R**)	0
		PB.9362	***bZIP11***	TanjilG_23462, Lupan_006525	TF bZIP11-like	NLL-08	−5.21 (1.27e-12; **R**)	0	−3.50 (6.49e-07; **k**)	0
		PB.4338	***IAA4***	TanjilG_09391, Lupan_031517	auxin-induced protein 22B-like	NLL-11	0	0	−11.15 (1.06e-41; **S**)	−12.78 (2.10e-56; **S**)
		PB.7173	***MYB59***	TanjilG_03285, Lupan_014873	TF MYB59-like isoform X1	NLL-07	−3.25 (2.08e-04; **k**)	0	−2.60 (2.19e-03; **R**)	0
		Lupan_024589	***MYB108***	TanjilG_29228, Lupan_024589	TF MYB108-like isoform X2	NLL-17	−5.94 (7.47e-06; **k**)	0	−4.69 (1.80e-05; **k**)	0
		PB.15930	***RAP2.3***	TanjilG_27008, Lupan_033269	Ethylene-responsive TF RAP2–3-like	NLL-16	0	0	−3.14 (3.51e-05; **R**)	0
	Secondary metabolism and biosynthesis	Lupan_010622	***ARG2***	TanjilG_16486, Lupan_010622	Indole-3-acetic acid-induced protein ARG2-like	NLL-04	−4.14 (1.04e-04; **k**)	0	−3.05 (3.84e-03; **k**)	0
		Lupan_030034	***ATL42***	TanjilG_12559, Lupan_030034	E3 ubiquitin-protein ligase ATL42-like	NLL-14	−2.47 (1.60e-05; **k**)	0	−2.54 (1.49e-09; **k**)	0
		PB.17657	***CES15***	TanjilG_00283, Lupan_028114	Probable carboxylesterase 15	NLL-19	−5.40 (1.09e-10; **k**)	0	−5.02 (8.82e-14; **k**)	0
		Lupan_024256	***CYP71A1***	TanjilG_05596, Lupan_024256	Cytochrome P450 71A1-like	NLL-17	0	0	−7.59 (1.89e-32; **k**)	−8.35 (4.32e-28; **k**)
		Lupan_005954	***CYP81E8***	TanjilG_02640, Lupan_005954	Cytochrome P450 81E8-like	NLL-08	−5.81 (3.01e-03; **k**)	−5.40 (1.82e-04; **k**)	−9.25 (2.44e-05; **S**)	−4.12 (1.43e-03; **k**)
		PB.14161	***CYP82A4***	TanjilG_22427, Lupan_015682	Cytochrome P450 82A4-like	NLL-02	−3.11 (1.89e-05; **R**)	0	−3.09 (7.90e-09; **R**)	0
		Lupan_024909	***7DLGT***	TanjilG_25542, Lupan_024909	7-deoxyloganetic acid glucosyltransferase-like	NLL-17	−8.80 (9.58e-15; **R**)	−5.95 (5.89e-22; **R**)	−9.58 (3.04e-40; **R**)	−7.48 (8.07e-51; **R**)
		PB.19771	***MXMT1***	TanjilG_17310, Lupan_023417	7-methylxanthosine synthase 1-like	NLL-13	−3.86 (1.82e-08; **R**)	0	−3.49 (1.37e-11; **k**)	0
*Known QA biosynthetic and regulatory genes*	Secondary metabolism and biosynthesis	PB.3339	***CYP71D189***	TanjilG_29685, Lupan_032119	Cytochrome P450 71D10-like	NLL-11	0	0	0	0
		PB.10302	***HMT-HLT***	TanjilG_22250, Lupan_010746	13-hydroxylupanine O-tigloyltransferase-like	NLL-04	0	0	0	0
		Lupan_033908	***LaAT***	TanjilG_21586, Lupan_033908	Spermidine coumaroyl-CoA acyltransferase-like	NLL-16	−11.81 (1.95e-17; **S**)	0	0	0
		PB.16302	***LaCAO***	TanjilG_00530, Lupan_020242	Amine oxidase [copper-containing] zeta, peroxisomal-like [*Gastrolobium bilobum*]*	NLL-15	−16.15 (1.60e-35; **S**)	0	0	0
		PB.16972	***LDC***	TanjilG_09726, Lupan_020529	Ornithine decarboxylase-like isoform X1	NLL-15	−16.78 (1.30e-38; **S**)	0	0	0
		PB.16320	***SDR1***	TanjilG_00579, Lupan_020288	(−)-isopiperitenol/(−)-carveol dehydrogenase, mitochondrial-like	NLL-15	0	0	0	0
	Transcription and gene expression regulation	PB.7870	***RAP2–7***	TanjilG_07628, Lupan_013780	Ethylene-responsive TF RAP2–7-like	NLL-07	−4.25 (1.69e-05; **S**)	0	0	0

v1 IDs are TanjilG_xxxxx from the NLL genome annotation of Hane et al. 2017 [34] and v2 IDs are Lupan_xxxxxx from the NLL genome annotation of Garg et al. 2022 [33]. R—RSEM, S—StringTie, k—kallisto refer to the differential expression method used. An asterisk (*) denotes best hit obtained from RefSeq Viridiplantae in absence of annotated hits in *L. angustifolius* reference.

**Figure 4:**
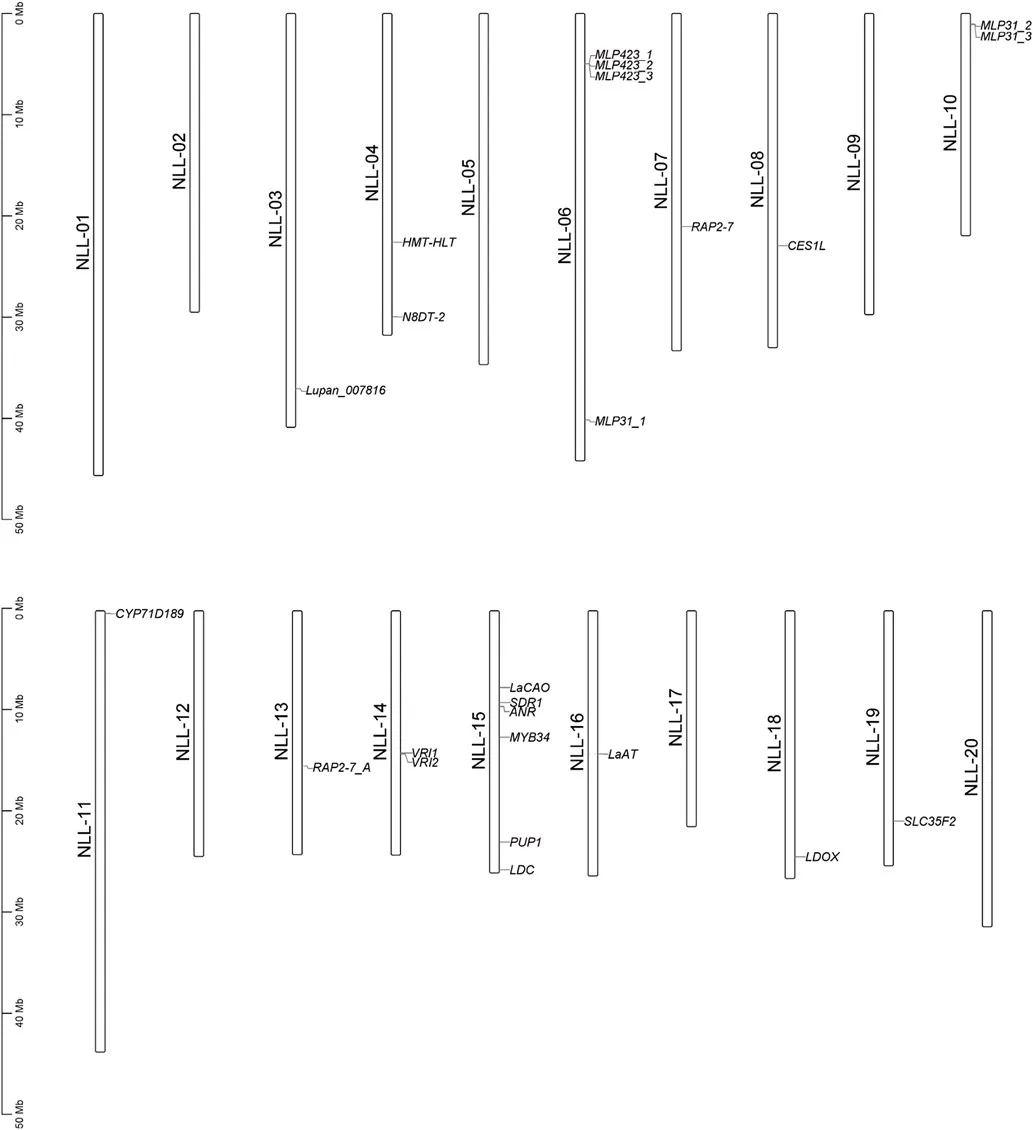
Chromosomal distribution of known QA pathway genes together with candidate genes from the *iucundus* versus *Iucundus* contrast in NLL. Map of the 20 NLL chromosomes in the *iucundus* reference genome [33] with chromosomes scaled in megabases and labels marking gene positions, generated using TBtools.

In *iuc* vs *Iuc*, candidates were mainly down-regulated in leaves but not stems, with fold change ranging from −23.40 to −5.52. In the reference set, *RAP2-7*, *LDC* (PB.16972), *LaCAO* (PB.16302), and *LaAT* (Lupan_033908) showed the same leaves restricted down-regulation. In contrast, *SDR1* (PB.16320) and *CYP71D189* (PB.3339), previously linked to *LDC* by co-expression [10], showed no consistent direction of change, and *HMT-HLT* (PB.10302) was not differentially expressed, consistent with prior reports [12]. These expression patterns are illustrated on Fig. S1 as RNA-seq heatmaps alongside a schematic overview of the currently known enzymatic steps of QA biosynthesis. In Bryansk vs *Iuc* contrast, all candidates were down-regulated in leaves and several in both organs, with fold change from −12.78 to −1.96. Across both contrasts, candidate genes were distributed among overlapping functional categories, with most assigned to secondary metabolism, transcriptional regulation, and additional representation in transport and ion homeostasis, protein sorting and ethylene biosynthesis (Table 2).

### Variant profiling in candidate and known QA-related genes

Variant discovery was performed using RNA-seq dataset. In the *iuc* vs *Iuc* contrast variant analysis was focused on candidate genes identified in this contrast and known QA-related genes. In the Bryansk lines the analysis was restricted to the known QA-related genes. Quality controlled reads mapped to the reference genome yielded 471 variants in these loci, including SNPs and small insertion–deletion variants (InDels) (Table S15). To assess the relevance of these polymorphisms, all 471 variants were further cross-referenced with the NLL pangenome panel (variants identified with respect to Tanjil baseline) contrasting 17 *iucundus* and 31 *Iucundus* accessions [33]. After Bonferroni correction, 118 variants differed significantly in allele distribution between the two groups (adjusted *P* < 0.05) and were detected in 16 genes. The highest number of differentially distributed variants were found in *RAP2-7* and *LDC* loci. Five significant variants were predicted to have a moderate impact and to alter the amino acid sequence, including two in *RAP2-7* and one each in *Lupan_007816*, *MYB34*, and *LDC*. The strongest support was obtained for variants in *RAP2-7*, including the one previously reported to be associated with the *iucundus* type [11, 12]. The Tanjil reference allele in *Lupan_007816* and *MYB34* was more frequent in low-alkaloid lines, whereas in *LDC* the alternative allele was more prevalent in low-alkaloid lines, including the three Bryansk lines.

### Focused analyses of selected candidate genes

Focused analyses examined *RAP2-7* and *RAP2-7A*, as well as *MLP* family candidates, in greater detail. *RAP2-7* and *RAP2-7A* were assessed by expression profiling, protein comparison and the previously mentioned pangenome variant analysis, whereas the *MLP* candidates were characterized at the protein domain level.

Across the analyzed organs, *RAP2-7A* expression closely matched the *RAP2-7* pattern (Fig. S2). The *iuc* vs *Iuc* difference was most evident in leaves, while in stems the two genotypes did not show significantly different expression levels for either gene. Sequence comparisons highlighted two distinguishing features. First, *RAP2-7A* retained arginine at the position corresponding to residue 196 in *RAP2-7* in both sweet and bitter genotypes, whereas *RAP2-7* differed at this position, with the sweet genotype carrying the R196S substitution (Table S15, Fig. S3). Second, comparison based on the low-alkaloid reference genome Tanjil showed that *RAP2-7A* encoded a shorter full-length protein than *RAP2-7*, with an 82 amino acid truncation in the C-terminal region (Fig. S3). In the pangenome panel, this same C-terminal position was polymorphic, and a stop loss variant was preferentially present across high QA accessions, although this association was not significant at the 0.05 threshold (*P* = 0.06). Moreover, at the protein level, the AP2/ERF DNA binding domain (IPR001471, PF00847) was highly conserved between RAP2-7 and RAP2-7A, with 93.75% identity.

All MLP candidates shared the signature for Bet v I/major latex protein domain (IPR000916, PF00407), which accounts for most of the protein length (Fig. S4). Further analysis revealed two distinct subgroups corresponding to IPR052006 (Major Latex-like) and IPR051761 (MLP-like hydrophobic ligand-binding) signatures. These exhibit low intergroup sequence similarity (averaging 25.2%) and corresponded to two different PANTHER database matches. Intergroup differences involve additional conserved sequence features associated with respectively: a Major Latex-like region and a MLP-like hydrophobic ligand-binding region. Domain alignments showed moderate to high similarity within each subgroup (45.8%–93.3%).

### Mapping of RAP2-7 TFBS

DAP-seq was focused on *RAP2-7*, the previously established candidate regulatory gene at the *iucundus* locus, providing the strongest rationale for targeted characterization [7, 11, 12]. All DAP-seq libraries passed initial quality control, with fractions of reads in peaks (FRiP) in 7.8%–13.6% range for all samples, however upon greenscreen filtering of artifactual signals, lower final FRiP estimates of 0.1%–1.5% could be seen [35], reducing the number of peaks from 2158 to 1130 (Table S16). Across genotypes, the number of inferred TFBS varied, with 15 sites in 83A:476 and 464 in P27255, compared with 54, 232, and 537 sites in 95826, 95927, and 95928, respectively.

Inferred RAP2-7 DAP-seq TFBS were distributed across intergenic regions (66%), exons (11%), introns (11%), promoters (7%), and transcription termination regions (5%). Among the intergenic sites, 62% were positioned more than 10 kb upstream/downstream of the nearest TSS. After excluding intergenic peaks, 383 sites were linked to their nearest gene based on promoter proximity, representing 370 unique genes, 256 of which had biological function annotation. Integration with RNA-seq showed that only 8.6% of the genes associated with TFBS were differentially expressed for at least one contrast, indicating that TF occupancy alone did not predict the direction of expression change.

### RAP2-7 regulatory motif reconstruction and docking simulation

Using conservative greenscreen peak filtering, *de novo* motif discovery in P27255 identified a centrally enriched AP2/ERF-like RAP2-7 binding motif, with the consensus sequence GTACGAGGTTT (top MEME result) (Fig. 5). Notably, no groups of centrally enriched motifs were recovered for 83A:476. In the Bryansk lines the same main motif was recovered with genotype dependent prevalence across peaks, corresponding to CentriMo motif group with up to 76% occurrence in 95826. On comparison with JASPAR2024 reference motif database, significant similarities to SMZ (AT3G54990), TOE2 (AT5G60120), and RAP2-7 (AT2G28550) binding site motifs from *A. thaliana* were detected (Fig. 5B) [36].

**Figure 5:**
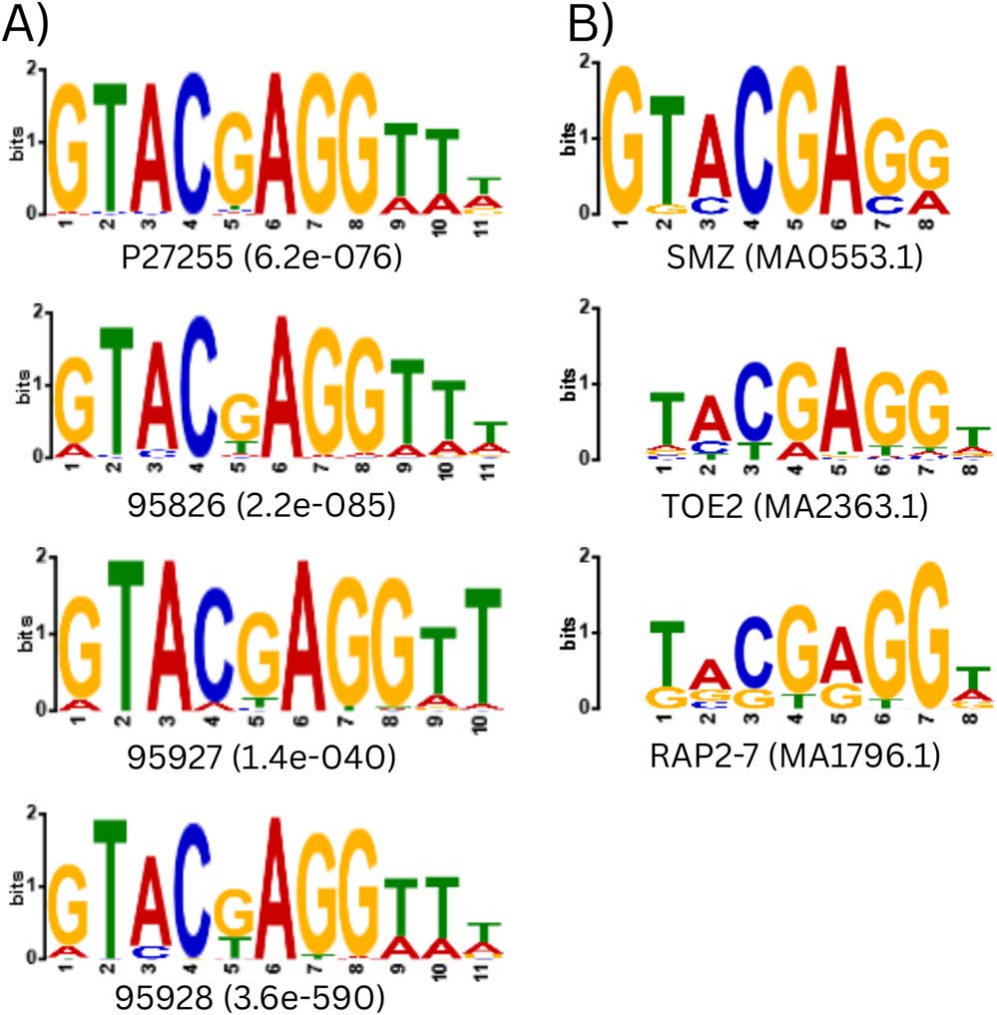
Comparison of RAP2-7 DAP-seq motifs across NLL genotypes and Arabidopsis homologs: (A) sequence logos for RAP2-7 DAP-seq binding motifs in four NLL genotypes; (B) known motifs of Arabidopsis SMZ, TOE2, and RAP2-7 retrieved from JASPAR (MA0553.1, MA2363.1, MA1796.1).

Previously, we have demonstrated that the arginine to serine substitution (R196S, relative to P27255 sequence) in NLL *RAP2-7* is strongly associated with low-alkaloid content [11]. To examine whether this mutation alters DNA motif recognition, we compared AlphaFold3 models of Tanjil RAP2-7, which carries the low-alkaloid S196 variant, and the alternative R196 version corresponding to high-alkaloid accessions, each bound to the reconstructed motif as DNA duplex. Although both structure models were of low quality (pTM = 0.35 for the Tanjil reference and pTM = 0.33 for the R196 variant), the AP2/ERF domain core (residues 149–211) was considerably more accurate (pDDT≥70). Accordingly, the model provides support for local contacts within this higher confidence AP2/ERF domain core, rather than the global arrangement of the full RAP2-7:DNA complex. Prediction of hydrogen bonds based on these models indicates that, within the domain core, R196 facilitates formation of a hydrogen bond with the second thymidine of the motif (T2). Its additional contacts with adjacent residues help stabilize the RAP2-7:DNA complex, whereas in the S196 variant this T2-centered interaction network is disrupted (see Fig. 6).

**Figure 6:**
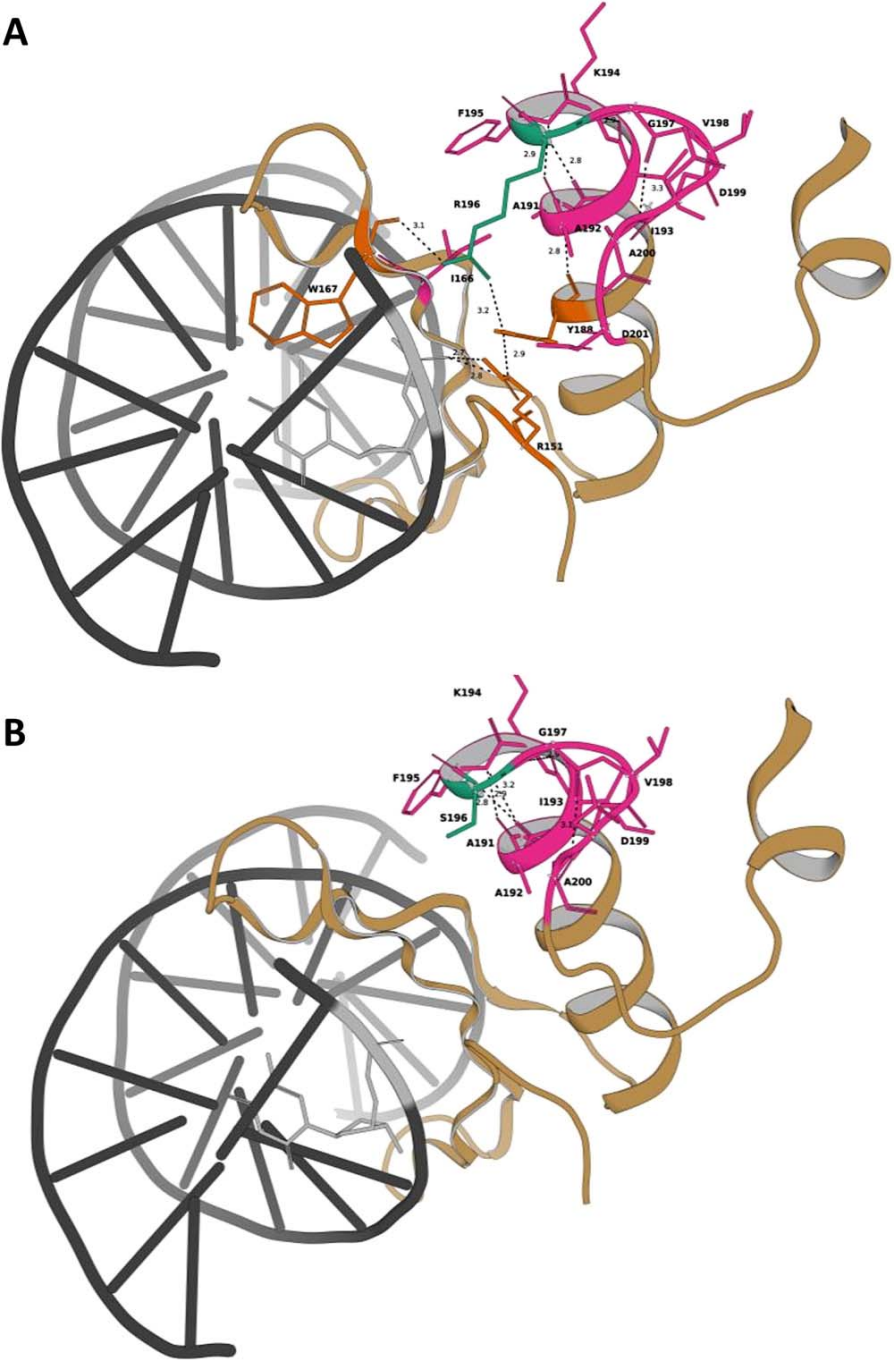
Residue R196 interactions in RAP2-7 variants in complex with DNA. (A) R196 in RAP2-7, representing the *Iucundus* allele, forms a hydrogen bond with T2 of the motif. (B) S196 in RAP2-7, representing the *iucundus* allele, shows no clear network of hydrogen bonds to T2 of the binding motif. Residue 196 is shown in dark green. H-bonding partner residues in the vicinity of DNA are shown in orange, and additional R196-proximal protein residues interacting with its local environment are rendered in magenta. DNA is depicted in dark gray, with bases directly adjacent to the R196 microenvironment shown in a lighter gray. Distances marked over putative bonds are in angstroms (Å).

### Integration of genome-wide RAP2-7 motif scanning with DAP-seq and differential expression

To better assess RAP2-7 binding, and to account for both DAP-seq sites that may have fallen below peak-calling thresholds and possible loss of binding competence due to the R196S substitution also present in the low-alkaloid NLL reference genome, cv. Tanjil [33], we used a two-part strategy.

First, using the P27255 RAP2-7 reference motif, we compiled a comprehensive set of matching loci across NLL genome and cross-referenced these with differential expression results and DAP-seq regions. At a relaxed *P*-value threshold of 1 × 10^−4^, the scan returned 96 060 motif occurrences, 8451 (8.8%) of which fell within 2 kb promoter regions. Of these, 684 occurred in association with DEGs (Table S17). For the *iuc* vs *Iuc* DEGs, promoter-level contingency counts were: 515 DEGs with a motif, 37 721 DEGs without a motif, 7312 non-DEGs with a motif, and 30 924 non-DEGs without a motif. Fisher’s exact test indicated strong depletion of the RAP2-7 motif among promoters of DEGs (odds ratio of ~0.058, *P* < 2.2 × 10^−16^). For the Bryansk vs *Iuc* comparisons, promoter-level counts were: 527 DEGs with a motif, 37 709 DEGs without a motif, 7300 non-DEGs with a motif, and 30 936 non-DEGs without a motif. Fisher’s exact test again indicated strong depletion of the RAP2-7 motif among promoters of DEGs (odds ratio of 0.059, *P* < 2.2 × 10^−16^).

Second, promoters of known and candidate QA-related genes (Table 2) were further inspected in this regard, yielding 14 putative TFBS across proximal regions of 13 genes, including 12 DEGs and one non-differentially expressed known gene *CYP71D189* (Table 3). Among the DEGs, two were *iuc* vs *Iuc* candidates, eight were Bryansk vs *Iuc* candidates, and two were known QA-related genes. Strong matches were identified in particular in the promoters of the *RAP2-7* itself, the known QA gene *LaAT* and the candidate *bZIP11* TF (PB.9362). The RAP2-7 binding motif identified in the *LaAT* promoter was further examined in the NLL pangenome panel. At its second position, low QA accessions carried cytosine almost exclusively (same as in the low-alkaloid reference cultivar Tanjil), whereas high QA accessions included both allelic states, with thymine present in 12 of 31 lines (Table S15).

**Table 3:** RAP2-7 binding motif occurrences in 2 kb promoters of candidate and known QA related genes.

Candidate gene set	Gene ID (current)	Gene symbol	NLL chromosome	Upstream coordinates	*P*-value	Matched sequence
*iucundus* vs *Iucundus* contrast	PB.14959	*LDOX*	NLL-18	−196:-186/+	0.0000954	TCACGAGGTTT
	PB.16702	*PUP1*	NLL-15	−1476:-1466/+	0.0000279	ATACTAGGTTT
Bryansk vs *Iucundus* contrast	PB.8060	*CAX3*	NLL-07	−1742:-1732/+	0.0000906	GTAAGAGGAAA
	PB.8060	*CAX3*	NLL-07	−1236:-1226/+	0.0000592	GTAAGAGGTAA
	Lupan_007045	*ZIFL1*	NLL-08	−663:-653/+	0.0000379	GTTCGAGGTAG
	PB.14361	*ACS*	NLL-18	−1517:-1507/+	0.0000443	GTGCGAGGATA
	PB.9362	*bZIP11*	NLL-08	−1771:-1761/+	0.0000041	GCACGAGGTAG
	PB.4338	*IAA4*	NLL-11	−1189:-1179/+	0.0000741	GTCCAAGGAAG
	Lupan_024589	*MYB108*	NLL-17	−1286:-1276/−	0.0000675	GTAGGAGGATA
	Lupan_010622	*ARG2*	NLL-04	−1522:-1512/+	0.0000715	GTTCGAGGAAG
	PB.19771	*MXMT1*	NLL-13	−857:-847/−	0.0000823	ATACAAGGATA
Known QA biosynthetic and regulatory genes	PB.3339	*CYP71D189*	NLL-11	−1025:-1015/+	0.0000906	GTATGAGGAAA
	Lupan_033908	*LaAT*	NLL-16	−902:-892/+	0.0000148	GCACCAGGTTT
	PB.7870	*RAP2–7*	NLL-07	−716:-706/+	0.000008	GTACCAGGTAA

Full FIMO results for the NLL genome are provided in Table S17.

Upstream coordinates indicate the start and end positions of the motif match within the scanned promoter, followed by strand orientation

## Discussion

### Integrative QA pathway insights in *iucundus*-regulated genotypes

Structural QA biosynthetic genes were previously shown to differ in expression among leaves, stems and green pods of *iucundus* and *Iucundus* NLL genotypes [7, 15, 37]. Our qPCR and RNA-seq data similarly show that *RAP2-7* and QA-related genes differ most strongly in leaves and pods and only weakly in stems (Fig. 2, Table 2). Despite this, QA content in stems of 83A:476 remained low compared with P27255, with a less pronounced fold difference between genotypes than in leaves or pods (Table 1). These patterns suggest that, under our experimental conditions, stems show only weak transcriptional regulation tied to QA differences and may primarily contribute to transport and storage, although epidermis specific QA biosynthesis previously reported in aerial organs of NLL [8] may have been masked in bulk stem samples. Together, our observations indicate that leaf expression profiles provide the most informative basis for candidate gene selection in this study. The leaf DEG set from *iuc* vs *Iuc* contrast encompassed known QA biosynthetic and regulatory genes, and revealed new pathway candidates (Table 2). Consistently, the Lupin eFP Browser showed elevated expression of all but one candidate gene in the epidermis of QA-producing organs, in line with the profiles reported for *LDC* and *LaCAO* [8], further supporting their functional relevance.

Given the incomplete understanding of the QA pathway, the candidate gene set may help fill some of the remaining enzymatic-, regulatory-, transport-, and storage-related gaps. One notable group comprises six loci encoding major latex proteins (MLPs), members of the pathogenesis-related protein 10 (PR-10; Table 2). Of these, four *MLP* genes map to NLL-06, with three forming a tight ~12 kb cluster (*MLP423_1*/PB.2036, *MLP423_2*/PB.2037, *MLP423_3*/PB.2039), while the remaining two candidates co-localize on NLL-10 (Table 2, Fig. 4). Although first linked to plant defense, MLP/PR10 proteins are also recognized as enzymes in specialized metabolism, catalyzing reactions once considered spontaneous. Examples include condensation, isomerization, and cyclization steps in BIA (morphine) biosynthesis [38–41]. These findings may also be relevant to the QA pathway, particularly in light of recent models proposing that several reactions considered chemically possible without enzymatic assistance may nevertheless require catalysis *in vivo* to ensure stereochemical control and pathway fidelity [9]. Beyond catalysis, opium poppy studies indicate that some MLP/PR10 proteins bind alkaloids, potentially contributing to their storage or transport [42]. These observations, together with previous links between MLP genes and alkaloid accumulation in *Lupinus* and *Sophora* [37, 43], support MLPs as plausible contributors to QA metabolism.

Another subset of candidates includes two vestitone reductase-like genes (*VR1*/PB.19347, *VR2*/PB.19348) and one anthocyanidin reductase-like gene (*ANR*/PB.16323) (Table 2). Domain annotation classifies them as NAD(P) dependent dehydratase-like enzymes (IPR050425), placing them in the broader class of NAD(P)H dependent oxidoreductases. In a recent QA pathway model, the tetracyclic di-iminium intermediate is converted to sparteine through two successive reductions attributed to one or more unidentified reductases [9]. Given their predicted activity and QA-like expression profiles, *VR1, VR2* and *ANR* are plausible candidates for these unresolved reductive steps. Analogous NAD(P)H dependent reductions of cyclic iminium substrates have been reported for noroxomaritidine reductase in Amaryllidaceae alkaloid biosynthesis [44]). Interestingly, although *VR1, VR2,* and *ANR* were annotated as flavonoid-related, such *in silico* classifications may underestimate their broader roles in specialized metabolism, including alkaloid biosynthesis [45–47]. Notably, *VR1* and *VR2* map to NLL-14 region spanning ~105 kb. *ANR* is located on NLL-15, within a ~5 Mb interval that also harbors *La*C*AO*, *SDR1* and the candidate regulator *MYB34* (Fig. 4).

Our data also point to an expanded regulatory component of the pathway, highlighted by a second *RAP2-7*-like TF on NLL-13, hereafter referred to as *RAP2-7A* (PB.20273; Fig. 4). *RAP2-7A* was previously reported as differentially expressed [12] and in our data showed even greater fold change, with an organ-specific expression pattern similar to *RAP2-7*. Across the Tanjil reference and pangenome panel, *RAP2-7A* showed sequence features inconsistent with a simple association with low QA phenotype, suggesting a more complex relationship between *RAP2-7A* variation and phenotype than implied by the R196 state alone. A further regulatory candidate was *MYB34*, for which pangenome-based variant analysis identified an amino acid substitution significantly associated with TAC (*P*-value <0.05). Together, these findings expand the set of potential regulators implicated in the QA pathway.

Last but not least, our candidate set also includes *CES1L* (Lupan_007209), previously linked to a stable QTL for isolupanine [12] and transport-related genes that may contribute to alkaloid translocation and sequestration (Table 2). Carboxylesterase-like enzymes contribute to specialized metabolism, including BIA and MIA biosynthesis, where they mediate unconventional catalysis and ring-forming cyclization [48, 49]. Among the transporter candidates, *PUP1* (PB.16702) maps near *LDC* on NLL-15 (Fig. 4), with homologous transporters mediating alkaloid movement and providing indirect biosynthetic pathway control in other species [28, 50]. Among the top DEGs in our contrasts, we also identified *SLC35F2* (PB.17620), a conserved solute carrier implicated in micronutrient uptake and drug transport in nonplant systems [51, 52].

### Insights into factors influencing QA metabolism in Bryansk lines

In our previous work [11], we demonstrated that Bryansk lines have low seed TAC despite carrying an *Iucundus*-type RAP2-7 allele, and showing high expression of QA-related genes in leaves, which argues against an *iucundus*-type mechanism of QA content regulation. We therefore considered alternative explanations, including structural gene mutations, impaired leaf to seed QA transport or altered expression of unknown QA genes.

In the current study, the Bryansk lines show a distinct QA chemotype, with lower proportion of lupanine and higher proportions of sparteine, isolupanine, and oxolupanine than both references (Fig. 1, Table S1). Elevated levels of upstream precursor (sparteine) [10], suggest a bottleneck in its conversion to lupanine, potentially accompanied by a rapid downstream turnover. Notably, RNA-seq showed no directional changes in *CYP71D189* and *SDR1* expression (Table 2), although missense substitutions in the N-terminal region of CYP71D189 may affect the predicted signal peptide and adjacent region (Table S15). Variant analysis, supported by the NLL pangenome, additionally showed that all Bryansk lines carry an amino acid substitution in *LDC*, whose significance for QA biosynthesis is difficult to interpret (despite its bitter origin, it was enriched in sweet accessions across the pangenome; *P*-value <0.05). Other variants in known QA genes are few and predicted to have low or modifier impact. These results suggest that neither coding disruption of known QA genes nor leaf to seed transport defects alone explain low TAC in the Bryansk lines, while the functional relevance of the detected variants remains to be resolved.

Organ level TAC and qPCR data, supported by the multiomics dataset, confirmed low QA content across Bryansk organs despite an *Iucundus*-type expression pattern of known QA genes (Table 1, Fig. 1), indicating that the core biosynthetic pathway is largely intact. GO enrichment identified fewer downregulated categories in Bryansk vs *Iuc* than in *iuc* vs *Iuc*, placing the Bryansk lines functionally closer to *Iucundus* (Fig. 3). In leaves, affected terms were enriched for xenobiotic and amino acid transport and responses to toxic substances, including *DTX21* (Lupan_008507) and *DTX16* (Lupan_018450), members of the MATE transporter family implicated in vacuolar sequestration of endogenous alkaloids in several species [53–55]. In stems, late endosome to vacuole transport was also weakened (Fig. 3), with lower expression of ESCRT components such as *CHMP1B* (Lupan_002219) and *BLOS1* (PB.3562) [56–58]. Given that the vacuole is the principal site of alkaloid sequestration [53, 54], these changes point to a bottleneck in vacuolar loading that may contribute to the low TAC observed across Bryansk organs. Although some changes may reflect a secondary adjustment to a smaller QA pool, the concordant transport-related shifts in leaves and stems support reduced detoxification and sequestration capacity in Bryansk lines.

The Bryansk lines also show reduced expression of several hormone-related genes (Fig. 3; Table 2), including *ACS* (PB.14361), related to ethylene biosynthesis, and *ZIFL1* (Lupan_007045), linked to basipetal auxin transport. RAP2-7 binding sequence motif matches were identified in both promoters (Table 3), suggesting a potential regulatory connection. Ethylene and auxin are known to modulate alkaloid biosynthesis in other species [59, 60]. Another auxin-related candidate, *IAA4* (PB.4338), showed no detectable transcript in the Bryansk lines, similar to *CHMP1B* (Table 2, Table S9). Additional downregulated candidates span transcriptional regulation, secondary metabolism, transport, and ion homeostasis (Table 2). Although their specific contribution to QA metabolism remains unresolved, these observations suggest that the Bryansk phenotype reflects additional regulatory or metabolic constraints alongside reduced intracellular trafficking and detoxification capacity.

### Towards a mechanistic view of RAP2-7 in QA biosynthesis

While our transcriptome-based analyses prioritized a broader set of candidates, DAP-seq and structural modelling focused on RAP2-7, previously established as the candidate regulatory gene at the *iucundus* locus [7, 12]. This focus provided a useful genetic reference point for interpreting regulatory differences across the contrasting genetic backgrounds, integrating sequence variation and expression data with DAP-seq to explore binding events and conserved motifs that were interpreted alongside sequence polymorphism and transcriptional variation. Comparative analysis revealed a pronounced depletion of high-confidence TFBSs in *iucundus* relative to *Iucundus* and Bryansk lines (Table S16). In line with this, *de novo* motif discovery recovered a strongly enriched, centrally positioned RAP2-7 binding motif from *Iucundus* and Bryansk peaks that closely matched the canonical SMZ, TOE2 and RAP2-7 recognition sequence [36] (Fig. 5), whereas no such motif was recovered from *iucundus* peak regions.

The *Iucundus* RAP2-7 binding signature is consistent with our previous phylogenetic analysis [12], which placed RAP2-7 (TanjilG_07628) and RAP2-7A (TanjilG_14185) in a well-supported legume subclade sister to the Brassicaceae AP2 proteins SMZ and SNZ and closely related to TOE2, thereby linking candidate-based genetic evidence with conserved AP2/ERF motif preferences. Promoter analysis further extended this integrative framework by identifying *in silico* matches to the recovered RAP2-7 motif in 13 candidate genes (Table 3). One example is *LaAT*, whose RAP2-7 consensus motif was retained across the *iucundus* and *Iucundus* pangenome panel, but varied at position 2, a site predicted to interact with RAP2-7 R196. The reference allele predominated among the low-alkaloid accessions, whereas the alternate T2 allele, which more closely matched the recovered RAP2-7 consensus motif, was more frequent in a subset of high QA accessions. These observations suggest that this promoter polymorphism may modulate RAP2-7-dependent regulation of *LaAT,* perhaps alongside the reduced activity associated with the R196S substitution in RAP2-7. More broadly, the absence of RAP2-7 DAP-seq peaks at candidate loci, together with the limited correspondence between predicted motif occurrence and differential expression across DEGs, suggests that RAP2-7 acts in a context-dependent manner and may involve indirect action through intermediate factors (such as RAP2-7A, MYB34, MYB108, or bZIP11), or distal binding. However, such modes of regulation still require further validation. It is also important to note that motif presence alone may not be sufficient to ensure detectable binding events under our assay conditions. This is consistent with large scale *A. thaliana* cistrome analyses and methodological assessments of DAP-seq, showing that sequence motif occurrences greatly exceed experimentally defined TFBS and that binding specificity depends on DNA context, motif sharing within TF families, chromatin context and cofactors [31, 32, 61, 62]. Consistent with studies showing that TF complexes can alter genomic binding profiles and motif usage [63, 64], RAP2-7 binding *in vivo* may likewise depend on yet unidentified partners. A relevant precedent for distal binding is tomato GAME9, an AP2/ERF factor that controls glycoalkaloid biosynthesis via a MYC2–GAME9 complex bound to a distal enhancer within a defined chromatin domain [65]. Co-expression network analysis, such as WGCNA, could complement DAP-seq in future studies by placing RAP2-7 within a broader transcriptional context, but robust module to trait associations would require a larger genotype panel than available here.

It is further important to consider that all DAP-seq reads were mapped to the NLL reference genome Tanjil [33], which carries the *iucundus* allele. Consequently, *Iucundus*-specific TFBS weakened, lost or altered in this background may be underrepresented, reducing peak detection and motif recovery. Similar patterns of TFBS decay and remodeling following reduced TF activity have been reported [66, 67]. Nevertheless, beyond these potential *cis*-regulatory differences, the reduced number of high-confidence peaks and the failure to reconstruct a clear, centrally enriched RAP2-7 motif for the *iucundus* allele are also consistent with reduced RAP2-7 binding caused by the R196S substitution. *In silico* modelling of RAP2-7 bound to DNA containing the reconstructed motif (Fig. 6) supports this interpretation and provides mechanistic insights into the association between RAP2-7 polymorphism and the low-alkaloid phenotype. More broadly, this study provides the first DAP-seq dataset for lupin, and thus establishes an initial benchmark for species-specific assay performance, including peak saturation and sensitivity.

## Conclusion

This study captured two distinct genetic architectures underlying low-alkaloid NLL phenotypes, prioritized candidate genes for previously unresolved steps in QA biosynthesis, and defined global expression changes associated with a non-*iucundus* mode of regulation. Our results further refine RAP2-7 from a positional candidate to a mechanistically supported, context-dependent regulator of QA biosynthesis, with the R196S substitution emerging as an important contributor to the low-alkaloid phenotype. Overall, this work refines the working model of RAP2-7 function and provides a coherent set of testable hypotheses and genetic targets for future functional studies and improvement of low-alkaloid germplasm. The associated datasets also establish new, high-quality transcriptomic resources and provide the first DAP-seq assay in NLL, elucidating characteristics of RAP2-7 binding sites.

## Materials and methods

### Plant material and sample collection

Five NLL accessions were analyzed. Two carry contrasting alleles at the *iucundus* locus: 83A:476 a domesticated sister line of the Wonga cultivar carrying the naturally occurring *iucundus* allele (low-alkaloid, also referred to as sweet or *iuc*) and P27255, a wild landrace from Morocco carrying the *Iucundus* allele (high-alkaloid, also referred to as bitter or *Iuc*) [68, 69]. The remaining three accessions, 95826 (Bryanskij-35), 95927 (Bryanskij-123), and 95928 (Bryanskij-237/83) show an alternative mechanism underlying low-alkaloid phenotypes and are referred to as the Bryansk lines according to their origin [11]. Detailed pedigree information for these accessions was not available in the accessible passport data.

In all datasets, each accession was analyzed individually. For interpretation and integrative comparisons across datasets, the three Bryansk lines were treated as a single group and compared with the *iucundus* and *Iucundus* regulatory types, collectively the references.

Plants were cultivated in a growth chamber (16 h photoperiod, 22/18°C day/night, 55%–60% relative humidity). Three independent cultivation experiments were conducted (2020, 2021, 2023), corresponding to successive stages of the work and covering all five accessions. Organs sampled for each assay block are detailed in Table 4. Samples collected for molecular analyses were flash frozen in liquid nitrogen and stored at −80°C, while samples for alkaloid assessment were processed fresh.

**Table 4:** Organs sampled and assays performed for five NLL accessions (83A:476, P27255, 95826, 95927, 95928).

Organ	qPCR, GC^1^	PacBio (Iso-Seq), Illumina (RNA-seq)^2^	DAP-seq^3^
Fully expanded young leaves	+	+	+
Stems (main and lateral)	+	+	
Flowers	+		
Green pods (pod walls, without seeds)	+		
Green, ripened seeds	+		
Dry, ripened seeds	+		

1-3: Each assay block was performed on organs collected within a single cultivation experiment.

### Transcriptome sequencing

#### RNA extraction

Total RNA was isolated from 30 mg of frozen, ground organ tissue (Table 4) using a Maxwell RSC Plant RNA Kit (Promega, Madison, WI, USA). RNA concentration and integrity were assessed with a NanoDrop spectrophotometer (Thermo Fisher Scientific, Waltham, MA, USA) and an Agilent 2100 Bioanalyzer (Agilent Technologies, Waldbronn, Germany), with a minimum RNA integrity number of 8. The same isolates were used to prepare PacBio Iso-seq and Illumina RNA-seq libraries.

#### PacBio Iso Seq library construction and SMRT sequencing

Total RNA from five plants per accession was pooled in equal amounts to prepare a single Iso-Seq library (Table S18). Libraries were constructed following PacBio’s Iso-Seq sequencing protocol combining nonsize selected and size selected fractions. Briefly, the first strand cDNA was synthesized using the SMARTer PCR cDNA Synthesis Kit (Clontech, CA, USA), amplified, and processed with the SMRTbell Template Prep Kit 1.0 (Pacific Biosciences, CA, USA). Sequencing was performed on the PacBio Sequel II system (HiFi/CCS mode; ≥20 GB raw data per sample) at Novogene Co. Ltd.

#### Illumina RNA-Seq library construction and sequencing

cDNA libraries for Illumina RNA-seq were prepared separately for each replicate (Table S18). mRNA was purified with oligo (dT) magnetic beads, fragmented, and reverse-transcribed with random hexamer primers, followed by second-strand synthesis. Libraries were prepared through end repair, A-tailing, adaptor ligation, size selection, amplification, and purification. Sequencing was conducted on the Illumina NovaSeq platform (PE150) at Novogene Co. Ltd.

### Transcriptome analysis

#### Consolidated annotation of NLL reference

Merged reference annotation combined the draft Ensembl/Plants release 60 annotation [34] with the updated chromosome-length reference [33], accessed from LupinExpress on October 10 2024, with additional annotation from Prof. Lingling Gao (personal communication). Consensus annotation was generated with LiftOver v1.6.3 [70] using coverage ≥0.5, child feature identity of ≥0.9 (−s 0.9), CDS polishing (−polish), and CDS status flags (−cds), with mappings provided by minimap2 v2.28-r1209 [71]. The resulting GFF3 files were merged using gffutils, retaining features from the newer assembly and adding a qualifier with previous identifiers to overlapping records (Dataset S1).

#### Reconstruction and curation of long-read transcriptome

Circular consensus sequences were generated using *pbccs* version 6.4.0 (minimum predicted accuracy 0.9). Primer removal and demultiplexing were performed using *lima* 2.13.0 in IsoSeq mode with peek-based primer inference. Full length non chimeric (FLNC) reads with detectable poly(A) tails were generated using *isoseq3 refine* (v4.0.0) and *pbtk* (v3.5.0), merged with *pbmerge*, standardized by assigning a unified SM tag in the BAM header, reheadered with SAMTOOLS (v1.21) [72], and indexed with *pbindex*.

FLNC reads were clustered using *isoseq3 cluster2* (QV support), to generate a consensus BAM, aligned to the NLL reference genome with *pbmm2* (v1.17.0, ISOSEQ preset), and collapsed with *isoseq3 collapse* using clustered alignments plus the original FLNC BAM, preserving distinct 5′ exon structures.

The GFF file with collapsed high-confidence transcript models was curated using SQANTI3 v5.3.6 (*sqanti3_qc.py*) [73] using all available Illumina reads, FLNC transcript counts and the post-LiftOver consensus annotation. Filtered models were then rescued using *sqanti3_rescue.py*. The full-splice matches (FSM) were required to have 0%–59% adenine downstream of the transcription termination site. Non FSM transcript types were rescued if they met at least one of three criteria: (i) 0%–59% downstream adenine, no reverse-transcription switching detected (RTS_stage = FALSE), isoform expression ≥50, and expression ratio ≥ 0.15; (ii) the same adenine and RTS_stage criteria plus fully canonical splice junctions; or (iii) the same adenine and RTS_stage criteria plus minimum coverage of three long reads. Transcripts meeting any criterion were retained, and reference models were added when no overlapping long-read derived model was present. The final annotation of [33] assembly in GFF3 format is included in the Supplementary Materials.

#### Functional annotation

Functional annotation was performed with EnTAP v1.1.1 [74] searches using DIAMOND v2.1.8 [75] against NCBI/RefSeq Plants and UniProt/SwissProt (downloaded at 23/05/2024), and with eggnog-mapper v2.1.12 [76], using EggNOG 5.0 database [77] for ortholog inference. Annotations were manually curated to retain only general or plant-specific functional terms.

#### Differential gene expression

Illumina RNA sequencing data were processed with the nf-core *rnaseq* pipeline v3.18 [78] (10.5281/zenodo.14537300) using the final annotation. Short reads were trimmed with TrimGalore v0.6.10 and cutadapt 4.9 [79] at default settings, then mapped and quantified using three approaches: RSEM v1.3.1 [80] with STAR v2.7.10a [81], StringTie v2.2.3 [82] with STAR and kallisto v0.51 [83]. Outputs from all methods were imported into R with tximport v1.30.0 [84], length normalized and modelled in DESeq2 v1.42 [85], using a two-factor (genotype and tissue) generalized linear model with interaction. Likelihood ratios were performed by comparing full and reduced models. In pairwise comparisons, genes with baseMean ≥5,|log₂ fold change| ≥ 2 and adjusted *P*-value ≤0.01 were reported as significantly differentially expressed. Gene Ontology (GO) enrichment was conducted with topGO v2.54 [86] using Fisher exact test (α = 0.05) and parent–child weighting algorithm [87] to account for GO term hierarchy.

## Candidate genes selection

Differential expression profiling and candidate genes selection were performed separately for the contrasts *iuc* vs *Iuc* and Bryansk vs *Iuc*, with the three Bryansk lines analyzed jointly as one group. The primary pool for candidate’s selection comprised genes significant in all three methods, with a small number manually incorporated from the broader set of significant DEG (significant according to at least one method). Final selection was based on functional annotations, GO term enrichment, expression in QA-producing organs [8], expression fold change, or prior literature evidence. This strategy yielded a robust list of candidate genes for each contrast combining high-confidence consensus DEGs with a few well-supported genes that strict filtering might have missed.

## Protein sequence comparisons

Protein sequences of RAP2-7, RAP2-7A, and MLP family candidates were obtained from the refined transcriptome resource based on PacBio transcript models mapped to the Tanjil reference genome, and thus primarily reflect the low-alkaloid sequence background. Domain annotation was performed using web-based InterPro Search by sequence, accessed on 16 February 2026 [88]. Multiple sequence alignments were generated using Clustal Omega via the EMBL-EBI web interface, accessed on 16 February 2026 [89].

## Variant calling of candidate and QA-related genes

Trimmed reads from nf-core/rnaseq pipeline were re-aligned with HISAT2 v2.2.1 [90] to the Tanjil reference in downstream-transcriptome–aware mode, providing splice junctions from the final annotation. Alignments were converted to sorted, duplicate marked BAMs with Sambamba v1.1.0 [91]. Variant calling followed Genome Analysis ToolKit v4.6.2 [92, 93] best practices using AddOrReplaceReadGroups, SplitNCigarReads, and HaplotypeCaller with multithreaded pair-HMM and the –recover-dangling-heads option. Variants were annotated with Variant Effect Predictor v114.1 [94] to infer their impact on protein sequence and splicing sites. SignalP 5.0 [95] and Phobius [96] were used to assess whether variants detected in the N-terminal region of gene of interest fall within the predicted signal peptide.

For ascertaining the prevalence of candidate polymorphisms with respect to Tanjil baseline (referred to as “pangenome panel”), raw pangenome reads were downloaded from ENA for 48 NLL lines. The approach described above was adapted to genomic data with two key changes. Firstly, alignment was carried out using Minimap2 (using default preset for short reads). Secondly, GATK HaplotypeCaller was used in genomic variant calling mode (-ERC GVCF), individual genotype gVCFs were then merged using CombineGVCFs. Finally, joint variant calling was performed with GenotypeGVCFs. Prevalence of variants among high-alkaloid vs low-alkaloid accessions was tested using Fisher exact test with Bonferroni correction (R 4.2.5).

## *In vitro* Halo-Tag-RAP2-7 recombinant protein expression

### Constructing a gateway Halo-Tag-RAP2-7 expression clones

Halo-Tag-RAP2-7 constructs were generated by Gateway recombination cloning technology and the pIX-Halo destination vector (ABRC stock CD3–1742) carrying N-terminal Halo-tag. RAP2-7 coding sequence was amplified with gene-specific primers (F: 5′-CACCATGGCTATGTTTGATCTCAAT-3′, R: 5′-TCAACTTGGGGAGTTAAATGAGGA-3′), cloned into pENTR/SD/D-TOPO entry vector (Thermo Fisher Scientific), sequenced (Genomed S.A., Warsaw) and validated against the RAP2-7 reference sequence (RefSeq: XM_019579992.1).

### *In vitro* synthesis of Halo-Tag-RAP2-7 recombinant protein

Halo-Tag-RAP2-7 was synthetized *in vitro* using the TNT T7 Coupled Reticulocyte Lysate System (Promega) with 1 μg of plasmid DNA template (verified Halo-Tag-RAP2-7 clones) according to the manufacturer protocol. Negative controls without plasmid DNA template were also included. Five microliters of lysate were used for western blot analysis and the remaining volume was frozen at −80°C for DAP-seq.

### Western blot analysis

Samples were denatured, separated in PAAgels (10% acrylamide) and electroblotted for 35 min onto a 0.22-μm nitrocellulose membrane (Schleicher & Schuell, Dassel, Germany) using a semidry blotting apparatus (Bio Rad, Hercules, CA, USA). Membranes were stained with Ponceau red, incubated with Anti-HaloTag antibody (Promega; 1:1500) and HRP conjugated anti-mouse immunoglobulin G (Santa Cruz Biotechnology, Dallas, TX, USA; 1:5000) for 1 h each. Signals were detected with a WesternBright Quantum chemiluminescent substrate (Advansta, San Jose, CA, USA) and imaged using a G:BOX system (Syngene, Cambridge, UK). The expected size of Halo-Tag-RAP2-7 recombinant protein was ~81 kDa.

### DAP-seq library construction and processing

Genomic DNA library preparation and DAP-seq were performed largely according to [31]. The main steps and relevant experimental details are summarized below.

DAP-seq libraries were prepared in three biological replicates per genotype. High-molecular-weight genomic DNA was extracted from frozen young leaves (3 g) using a phenol:chloroform:isoamyl alcohol protocol, and quantified using Qubit (Thermo Fisher Scientific). An aliquot of 7.5 μg in EB buffer was then sonicated to ~200 bp fragments (Bioruptor Plus, Diagenode), size-selected with AMPure XP beads (2:1 bead: DNA ratio; Beckman Coulter Life Sciences, Indianapolis, IN, USA), end-repaired (Fast DNA End Repair Kit, Thermo Fisher Scientific) and purified (GeneJET PCR Purification Kit, Thermo Fisher Scientific). Next, samples were A-tailed (Klenow 3–5′exo-, New England Biolabs, Inc., Ipswich, MA, USA), purified again and ligated with truncated Illumina TruSeq Y-adaptors (T4 DNA Ligase, New England Biolabs). Finally, libraries were cleaned with AMPure XP beads (1:1), eluted in 40 μl of EB, and quantified with Qubit (Thermo Fisher Scientific).

Halo-Tag-RAP2-7 protein was immobilized on Magne HALO-Tag beads (Promega) (1 h, RT), washed five times with 1× PBS containing 0.005% NP 40 (PBS-NP40) and incubated with 1 μg of adaptor-ligated DNA library (1 h, RT). Negative controls were processed in parallel by incubating adaptor ligated DNA libraries from each accession with beads lacking recombinant protein. Protein–DNA complexes were washed eight times with PBS-NP40, bound DNA was eluted in 25 μl EB and amplified using Illumina TruSeq dual indexed primers (Primer A: 5’AATGATACGGCGACCACCGAGATCTACAC-NNNNNNNN-ACACTCTTTCCCTAC and Primer B: 5’CAAGCAGAAGACGGCATACGAGAT-NNNNNNNN-GTGACTGGAGTTCAG, where NNNNNNNN represents the i5/i7 index sequences). Final libraries were purified with AMPure XP beads (1:1), quantified with Qubit, and sequenced on an Illumina NovaSeq X Plus (PE150, Novogene Co. Ltd.), yielding ~6 Gb of raw data per sample (≥20 million reads per sample). Negative control libraries underwent the same procedure and were used to filter background peaks during peak calling.

### DAP-seq data analysis

DAP-seq data were processed with the nf-core chipseq pipeline v2.1.0 [78] (10.5281/zenodo.13899404), adapted to this assay. Short reads were trimmed with TrimGalore v0.6.7 and cutadapt v3.4 [79], aligned with Bowtie2 v2.5.2 [97] and peaks called with MACS3 v3.0.1 [98] in narrow peaks mode (FDR 0.01, genome size 613189654, read length 150), restricting analyses to chromosome scaffolds. Peaks were annotated with HOMER v4.11 [99] *annotatePeaks.pl* script. The resulting peaks were filtered against a blacklist obtained from MACS3 reanalysis of all controls following the “greenscreen” procedure [35], as peak areas supported by at least half of the samples. Centrally enriched consensus motifs were identified with MEMECHIP v5.5.8 [100], against genomic sequence masked using RED [101], and compared to the JASPAR 2024 plants nonredundant TFBS subset [102].

To ascertain presence of additional RAP2-7 TFBS, not detected by DAP-seq, NLL genome was additionally investigated with FIMO v5.5.8 [103] at *P*-value threshold of .0001 and using background frequencies derived from RED-masked genome. Association between the distribution of RAP2-7 motif and differential gene expression was assessed with Fisher exact test as implemented in R (v4.3.1).

## Structural modelling of RAP2-7:DNA complexes

Approximate models were created using AlphaFold3 [104] webserver with RAP2-7 protein sequences differing in alkaloid context-associated polymorphism (S196R, based on Tanjil reference NCBI/RefSeq protein XP_019435537.1) [11] and homodimer DNA corresponding to the majority rule consensus of the P27255 predicted STREME [105] motif (AGTACGAGGTTT). Structure visualization was done in PyMol v3.1.0 (The PyMOL Molecular Graphics System, Schrödinger, LLC) and hydrogen bonds predicted by HBPLUS [106].

## Gene expression analyses (qPCR)

Total RNA was extracted as detailed in section 2.1, and 1 μg was reverse transcribed with the Transcriptor First Strand cDNA Synthesis Kit (Roche, Mannheim, Germany) using anchored oligo (dT)18 primers. Expression of four alkaloid-related genes: APETALA2/ethylene response (AP2/ERF) TF (*RAP2-7*, TanjilG_07628) [12], lysine decarboxylase (*LDC*, TanjilG_09726) [3], copper amine oxidase (*LaCAO*, TanjilG_00530) [14], acyltransferase-like gene (*LaAT*, TanjilG_21586) [13] was quantified as described in [12] and normalized to *ADH3*, *TUBA*, and *ELF1B* according to [7]. In addition, expression of RAP2-7A, a candidate from this study, was quantified using the same qPCR workflow, cDNA set, and normalization strategy as used for RAP2-7, allowing direct comparison between the two transcripts. Relative expression was calculated with the efficiency corrected method [107].

## Alkaloid extraction and quantification

Quantitative and qualitative QA profiles were evaluated in three biological replicates per accession across six organs (Table 4). Alkaloids were measured by gas chromatography (GC-FID, GC-2014; Shimadzu, Kyoto, Japan), using the calibration equation *y* = 0.0159*x*, where *x* is the ratio of lupanine to caffeine peak areas and *y* is lupanine content, (mg). Extraction and analysis were performed as described previously [17, 18]. TAC was expressed as % seed dry weight, and individual alkaloids were quantified by their relative abundance, as percentage of TAC (defined as 100%).

## Data Availability

All genomic and transcriptomic data, generated in this study are available at ArrayExpress (EMBL-EBI) under the following accession numbers: E-MTAB-16429 for PacBio data, E-MTAB-16431 for Illumina RNA sequencing data, and E-MTAB-16430 for DAP-seq data.

## Supplementary information

Supplementary data supporting this study, including Figures S1–S4, Tables S1–S18 and Dataset S1, are available on Figshare under DOI 10.6084/m9.figshare.30739052.
